# Mr. Paget’s Report Continued

**Published:** 1844-05-18

**Authors:** 


### RECORD OF MEDICAL SCIENCE.

### MR. PAGET'S REPORT CONTINUED.

*Formation and structure of the membranes, &c.* M. Serres, in a paper read before the Institute of France, and in subsequent discussions, has maintained the view of Pockels, that the embryo is outside the amnios to the fifteenth or twentieth day, and that the amnios up to this time is a free vesicle, in which the embryo dips and envelopes itself (exactly as the ovum is supposed to envelope itself in the decidua) in a double sac. He adds, further, his belief that the allantois of the human embryo, having its pedicle immediately in front of the caudal prolongation, and at a distance from that of the umbilical vesicle, cannot be regarded as produced by a retroversion of the intestine, but has its origin in the corpora Wolffiana, whose existence in the human embryo he considers he has fully demonstrated. His view was supported by preparations, but in the discussions which followed the reading of the memoir, MM. Coste and Velpeau maintained each his own previous view of the matter, and said that the preparations did not demonstrate that of M. Serres.

Mr. John Dalrymple has described and figured the minute vessels of the vitelline membrane and allantois of the chick. Of the vitelline membrane he says, that immediately around the remains of the vitelline area are seen on the internal surface of the yelk-sac the commencement of a series of radiating folds, which as they advance dip deeper and deeper into the interior of the sac, and separate more widely from each other. When the vitelline cells are completely removed, it is seen that vessels alone constitude the framework of these folds, the large trunks forming their bases, while innumerable lesser branches dip deep into the interior of the sac, inosculating repeatedly, and terminating in a series of very tortuous branches, which fringe the extreme edge of each fold. Numerous simple loops are observed shooting from the sides of the larger trunks; and if we conceive each trunk and every small vessel thickly covered with an aggregated arrangement of vitelline globules or nucleated cells, which conceal the vessels and colour them bright yellow, we shall have a true idea of the appearance of these folds previous to the manipulation necessary to display the injection.

In the allantois Mr. Dalrymple says there is a very minute distribution of equal-sized capillary vessels throughout its inner layer, forming an uniform vascular surface covering the large trunks as well as the interspaces of their divisions; and the anastomoses of the capillaries are so numerous and close that the areas they leave do not exceed the diameter of the vessels themselves. From the similarity of this arrangement of vessels to that found in the lungs of the frog, salamander, &c., he thinks evidence may be adduced for the supposed respiratory function of the allantois.

Mr. F. Renaud, confirming (as nearly all now do) E. H. Weber's description of the arangement of the vessels of the placenta, points out as a chief source of fallacy in the examination of these structures, the rapidity with which the villi of the chorion absorb water, and are distended and confused by it.

M. Elsaesser has found in 144 fœtuses either born dead or living only a month, that in fifty-two born dead (of which forty-eight were mature and four immature), the ductus arteriosus, ductus venosus, and foramen ovale were all open forty-eight times. In four (one immature) the foramen ovale was closed, the others open.

In ninety-two dying in the first four weeks (of which twenty-two were premature) all the passages were open in fifty-eight. In eighty the foramen ovale was open; in seventy-seven the ductus arteriosus; in sixty-five the ductus venosus.

The most common mode of closure is: 1. The ductus venosus, beginning at the vena portæ. 2. The ductus arteriosus beginning at the middle. 3. The foramen ovale by the application of its edges. Even later than four weeks any of them may sometimes be found partly open.

### LACTATION.

M. Mandl confirms the view of Henle and others in regard to the perfect milk-corpuscles, proving the existence of an external membranous envelope by rubbing the corpuscles between glasses. The oil-globules are set free, and the torn membranes are unrolled and flattened.

M. Raciborski has examined the question of the influence of menstruation on the secretion of milk, and has found that it is unimportant. The only difference between the milk of nurses who do, and those who do not menstruate, is that in the former the proportion of cream is rather less in the menstrual period than it is in themselves in the intervals, and than it is generally in non-menstruating nurses.

### PHYSICAL HISTORY OF MAN.

*Characters of the Egyptian and Negro races.*—Dr. S. G. Morton has made observations on one hundred crania of ancient Egyptians, obtained at seven sepulchral localities from Memphis in Lower Egypt to Deboud in Nubia. He classes them as 1. *Arcto-Egyptians*, including the purer Caucasian nations, as seen in the Semitic tribes of Western Asia, and the Pelasgic of Southern Europe. 2. Austro-Egyptians, in which the cranium blends the characters of the Hindoo and Southern Arab; which people, the author thinks, were ingrafted on the original population of Ethiopia, and thus gave rise to the celebrated Meroite nations of antiquity. 3. Negroloid, in which the osteology of the crania corresponds to the Negro; but the hair, though harsh, is long and smooth, like the present Mulatto grades. 4. Negro.

The lines between these could not be exactly drawn. But in the one hundred skulls there might be reckoned fifty-six Arcto-Egyptians, twenty-eight Austro-Egyptians, six Semetic, seven Negroloid, one Negro, and two doubtful.

He deduces, therefore, 1. That Egypt was originally peopled by the Caucasian race. 2. That the great preponderance of heads like those of the purer Caucasians suggests that the valley of the Nile derived its primitive inhabitants from one of these sources. 3. That the Austral-Egyptian or Meroite communities were in great measure derived from the Indo-Arabian stock; thus pointing to a triple Caucasian source for the origin of the Egyptians, when regarded as *one people* extending from Meroe to the Delta. 4. That the Negro race exists in the catacombs in the mixed or Negroloid character: that even in this modified type their presence is comparatively unfrequent; and that if Negroes, as is more than probable, were numerous in Egypt, their social position was chiefly in ancient times what it now is, that of plebeians, servants, and slaves.

*Stature.*—Some very interesting observations on the stature of man have been made by Mr. A. Shaw. He shows that rickets not only produces softening of the bones, but arrests growth ; and this in the lower extremities much more than the upper, so that the child-like form, characterized by largeness of the head, trunk, and upper extremities, is persistent. These three parts are in persons stunted by rickets reduced only by 1-13 of the natural size; but the pelvis and lower extremities are reduced by 1-3. There is, therefore, an *arrest of developement* in regard to proportion of form.

This is shown further in that the proportion of size between the cranium and face remains as in childhood ; the former being always *proportionally* large, though *absolutely* not so large as in the well-formed adult. The proportions are in the child as 8 :1 ; in the well-formed adult as 6 : 1; in the rickety adult as 7 1-13 :1.

On the other hand, where growth is preternaturally active, as in giants, the lower half of the body is the part which is most increased, and it acquires disproportionate length. And in these, the cranium, though absolutely large, is, relatively to the face, small; e. g., in the skull of O'Byrne, the giant eight feet high, in the museum of the Royal College of Surgeons, the proportion of the size of the head to that of the face is only as five to one.

*Varieties of the pelvis.*—Dr. Knox, in his "Contributions to Anatomy and Physiology," shows that many or all the national peculiarities of the form of the female pelvis, as well as many of those which are regarded as malformations, are to be regarded as due to the fœtal form of the pelvis being more or less persistent. The fœtal form "is more quadrilateral than oval or rounded, and its antero-posterior diameter is the longest: it has the form, in great measure, of the pelvis of the quadruped and quadrumanous mammal, of the human male generally, and of of certain ill-formed human female pelves." When the persistence in this form exists on one side only of the pelvis, it produces the *pelvis ovata* of Naegele. Its more common effect when existing on both sides is to produce the not unfrequent narrow quadrilateral form of female pelvis ; but when it exists in an extreme degree on both sides, it may produce, as in a pelvis in Dr. Outrepont's collection, a kind of a double Naegele's oblique pelvis—one with a very long conjugate diameter, but very narrow in front—almost like a seal's pelvis.

He gives cases also of relaxation of the ligaments of the symphysis pubis in delivery.

